# Response to: ‘TARA Study: a new perspective on tapering drugs in RA’ by Mishra *et al*

**DOI:** 10.1136/annrheumdis-2019-215641

**Published:** 2019-05-20

**Authors:** Elise van Mulligen, Johanna M W Hazes, Angelique E A M Weel, Pascal H P de Jong

**Affiliations:** 1 Department of Rheumatology, Erasmus MC, Rotterdam, The Netherlands; 2 Department of Rheumatology, Maasstad Hospital, Rotterdam, The Netherlands

**Keywords:** DMARDs (biologic), DMARDs (synthetic), methotrexate, rheumatoid arthritis, treatment

We are pleased about the interest in our article by Mishra *et al* and we would like to respond to their questions so that there can be no ambiguity.^1 2^

First of all, there is some clarification needed on the conventional synthetic disease-modifying antirheumatic drugs (csDMARDs) that were used in combination with the TNF-inhibitors at baseline in the TApering strategies in Rheumatoid Arthritis (TARA) study. In table 1, we elaborate on the different combinations of csDMARDs that were used for each intervention arm separately. In the csDMARD tapering group, the methotrexate (MTX) was tapered, except for the three patients who did not use MTX. These patients gradually tapered leflunomide (n=1) and sulfasalazine (n=2).

**Table 1:** Use of csDMARDs at baseline in the TARA study specified for two groups: tapering csDMARDs and tapering TNF-inhibitors

Use of csDMARDs at baseline	Tapering csDMARD (n=93)	Tapering TNF-inhibitor (n=95)
MTX monotherapy, n (%)	64 (69)	49 (52)
MTX+hydroxychlorquine, n (%)	17 (18)	27 (29)
MTX+sulfasalazine+hydroxychloroquine, n (%)	5 (5)	6 (6)
MTX+sulfasalazine, n (%)	3 (3)	2 (2)
MTX+leflunomide, n (%)	1 (1)	0 (0)
Sulfasalazine monotherapy, n (%)	0 (0)	3 (3)
Sulfasalazine+hydroxychloroquine, n (%)	2 (2)	0 (0)
Sulfasalazine+leflunomide, n (%)	0 (0)	1 (1)
Leflunomide monotherapy, n (%)	1 (1)	3 (3)
Leflunomide+hydroxychloroquine, n (%)	0 (0)	1 (1)
Hydroxychloroquine monotherapy, n (%)	0 (0)	3 (3)

MTX, methotrexate; TARA, TApering strategies in Rheumatoid Arthritis; csDMARD, conventional synthetic disease-modifying antirheumatic drug.

Mishra *et al* also had a question about our intention-to-treat (ITT) analysis. In an ITT analysis, patients are analysed in the groups to which they were randomised, regardless of whether they received or adhered to the allocated intervention. Therefore, in the clinical response table of the original article, we should have given the total numbers instead of the patients who were still participating in the TARA trial at 12 months.^2^ If we had given the total numbers, the results would be similar.

Third question was about explaining the difference between the number of patients who are in remission after 12 months of follow-up and the number of patients below the Kaplan-Meier (KM) curve at 12 months. In a KM curve, only the patients at risk are given. Patients are censored if they experience a flare or drop-out, which results in a decreasing number of patients at risk over time. In the original TARA article, on the other hand, the number of patients in clinical remission (defined as a disease activity score (DAS) <1.6) at 12 months of follow-up is given. Thus, the interpretation of the numbers given in the KM curve and the number of patients in clinical remission is different and, therefore, the numbers are non-identical.

Finally, it would be interesting to know if the primary outcome would change if we use a modified per-protocol approach as brought up by Mishra *et al*. For this reason, we excluded the patients who used oral glucocorticoids, n=4 and n=5, respectively, in the csDMARD and TNF-inhibitor tapering group, or had more than one intramuscular injection, n=3 in each tapering group. With aforementioned approach a 30% (95% CI, 21% to 41%) flare rate was seen in the csDMARD tapering group, and a 39% (95% CI, 31% to 52%) flare rate in the TNF-inhibitor group (p=0.15). The difference in flare rates between the two tapering arms is similar to the one found in the original article.^2^
